# Risk factors for 2019 novel coronavirus disease (COVID-19) patients progressing to critical illness: a systematic review and meta-analysis

**DOI:** 10.18632/aging.103383

**Published:** 2020-06-23

**Authors:** Lizhen Xu, Yaqian Mao, Gang Chen

**Affiliations:** 1 Shengli Clinical Medical College, Fujian Medical University, Fuzhou 350001, Fujian, China; 2 Department of Endocrinology, Fujian Provincial Hospital, Fuzhou 350001, Fujian, China; 3 Fujian Provincial Key Laboratory of Medical Analysis, Fujian Academy of Medical Sciences, Fuzhou 350001, Fujian, China

**Keywords:** risk factors, COVID-19, systematic review, meta-analysis

## Abstract

Importance: With the rising number of COVID-19 cases, global health resources are strained by the pandemic. No proven effective therapies or vaccines for this virus are currently available. In order to maximize the use of limited medical resources, distinguishing between mild and severe patients as early as possible has become pivotal.

Objective: To systematically review evidence for the risk factors of COVID-19 patients progressing to critical illness.

Evidence Review: We conducted a comprehensive search for primary literature in both Chinese and English electronic bibliographic databases. The American agency for health research and quality tool was used for quality assessment. A meta-analysis was undertaken using STATA version 15.0.

Results: Twenty articles (4062 patients) were eligible for this systematic review and meta-analysis. First and foremost, we observed that elderly male patients with a high body mass index, high breathing rate and a combination of underlying diseases (such as hypertension, diabetes, cardiovascular disease, and chronic obstructive pulmonary disease) were more likely to develop severe COVID-19 infections. Second, compared with non-severe patients, severe patients had more serious symptoms such as fever and dyspnea. Besides, abnormal laboratory tests were more prevalent in severe patients than in mild cases, such as elevated levels of white blood cell counts, liver enzymes, lactate dehydrogenase, creatine kinase, C-reactive protein and procalcitonin, as well as decreased levels of lymphocytes and albumin.

Interpretation: This is the first systematic review exploring the risk factors for severe illness in COVID-19 patients. Our study may be helpful for clinical decision-making and optimizing resource allocation.

## INTRODUCTION

The World Health Organization (WHO) has declared COVID-19 a public health emergency of international concern (PHEIC) [1]. On January 7, 2020, the causative agent was identified as a novel coronavirus, which was later named 2019-nCoV [2–4]. According to the WHO report [5], as of the 11^th^ of April 2020, a total of 1, 610,909 confirmed cases and 99,690 deaths were reported globally. While the epidemic in China is gradually getting under control, America, Europe and the Middle East have all been facing rapid spread of the virus [5].

Nowadays, with the exponential increase in the number of infections, global health resources are extremely limited. In order to boost the repartition of limited medical resources, we ought to distinguish between mild and severe cases promptly. At present, no effective therapies or vaccines for this virus and the novel coronavirus pneumonia (NCP) are available. Moreover, our assessment ability for risk factors of patients developing severe pneumonia is limited. In this regard, we summarized the published studies conducted with critically ill patients to identify risk factors of NCP and provide Chinese experience to clinicians around the world on responding to COVID-19.

## RESULTS

**Figure 1:**
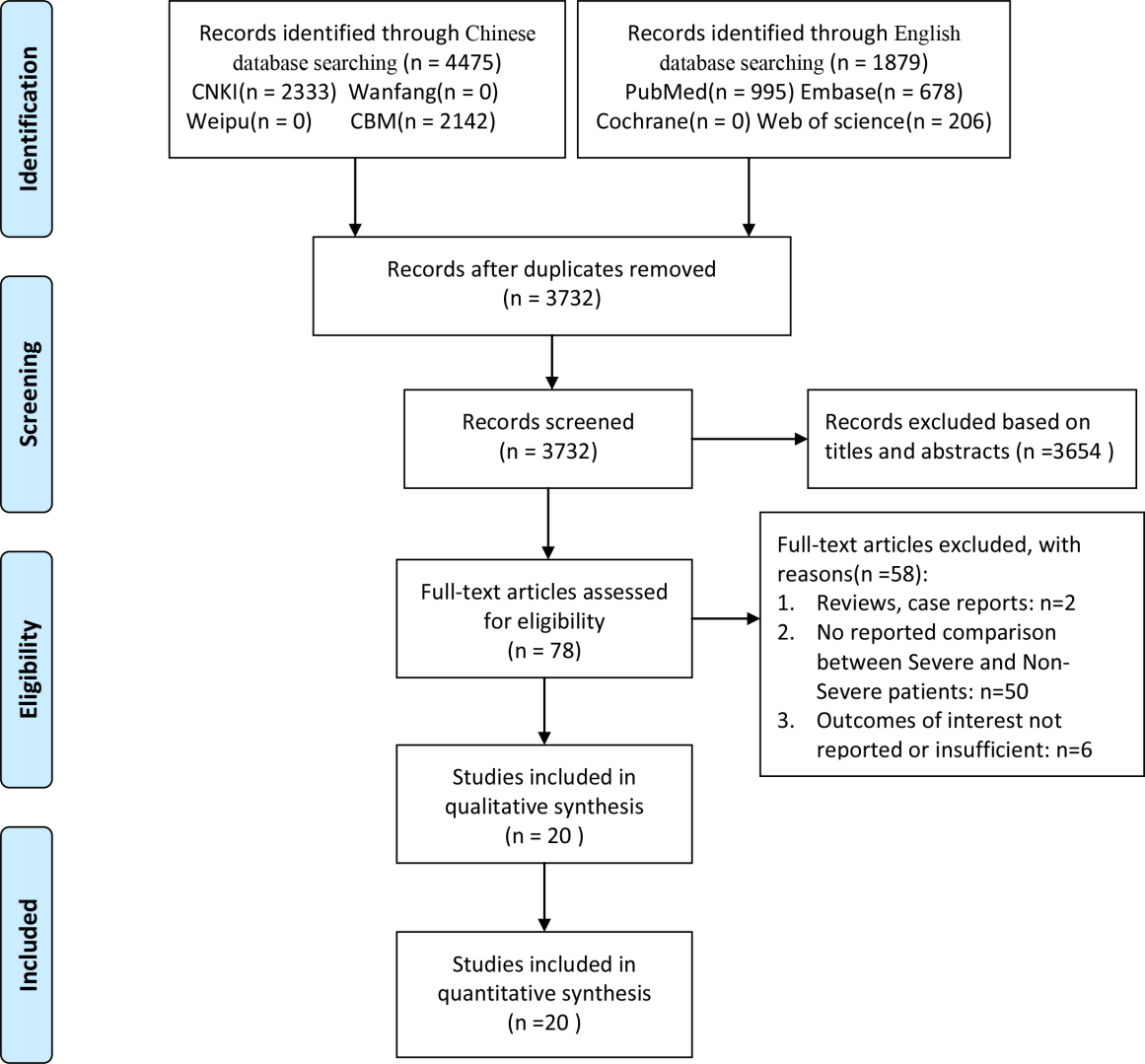
**A schematic flow diagram of studies’ search and retrieval process.**

The entire process of literature collection and screening is illustrated in Figure 1. Initially, 6354 publications were identified through database probing. After the exclusion of 2622 duplicates, there remained a record of 3732 studies. We eliminated 3654 records by reviewing their titles and abstracts. As a result, only 78 articles were subject to a full-text review. Finally, 20 articles [6–25] meeting the inclusion criteria were involved in the analysis.

**Figure 2:**
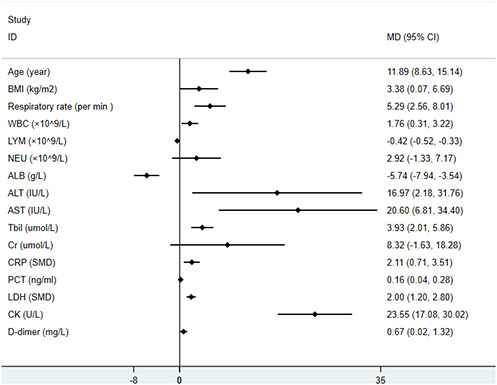
**The forest-plots of risk factors with COVID-19 patients on continuous variable.**

**Figure 3:**
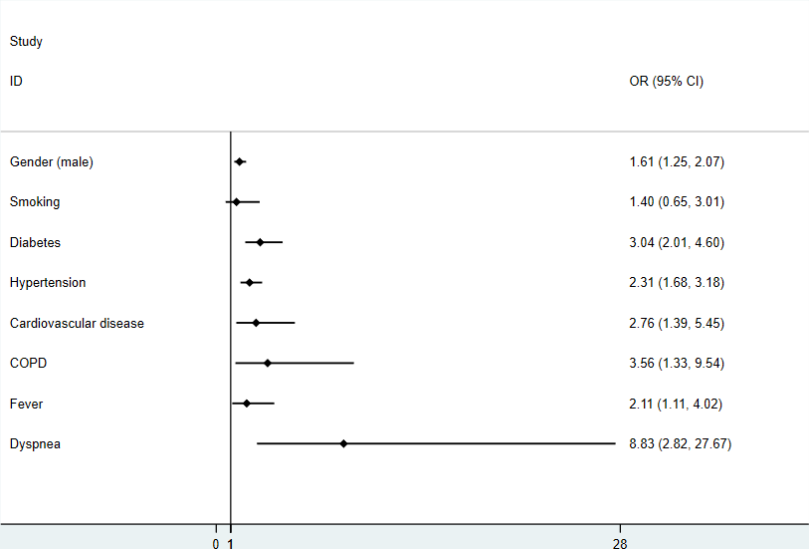
**The forest-plots of risk factors with COVID-19 patients on binary variable.**

Main characteristics of the studies were summarized in Supplementary Table 1. The results of the meta-analysis were displayed in Table 1. More intuitive results can be depicted in the forest-plots (Figure 2, Figure 3). All articles were observational studies. All subjects were from China, including more than 30 provinces and cities. The study period spanned from the 11^th^ of December 2019 to 23^rd^ of February 2020. Regarding the comparison between mild and severe patients, 15 studies [6–9, 11, 13–15, 17–20, 23–25] delineated patient characteristics, 15 studies described comorbidities [6–11, 13–17, 20, 23–25], 8 studies [6, 7, 13, 17, 19, 20, 24, 25] outlined vital signs, 17 studies compared symptoms [6–10, 13–20, 22–25] and 19 studies [6–18] presented laboratory findings. The study of Guan et al [6] (including 1099 patients from 552 hospitals in 30 provinces) explained the clinical characteristics of mild and severe patients in detail. Given the fact that the patients included in this article might overlap with other studies, and the results were principally expressed using median and quartile intervals, we did not include this study in our meta-analysis, and mainly used the method of descriptive analysis to compare the results with our studies.

**Table 1:** The meta-analysis of risk factors for severe patients with COVID-19

**Risk factor**	**Definition**	**Number of studies**	**Size (n)**	**OR/WMD/SMD (CI 95%)**	***I^2^***
**Patient characteristics**					
Age (year)	Continuous	6	681	11.89 [8.63, 15.14]	54.40%
Gender	Male vs. Female	10	1494	1.61 [1.25, 2.07]	0%
BMI (kg/m^2^)	Continuous	2	79	3.38 [0.07, 6.69]	67.20%
Smoking	Yes vs. No	3	412	1.4 [0.65, 3.01]	0%
**Comorbidities**					
Diabetes	Yes vs. No	10	1083	3.04 [2.01, 4.60]	20.40%
Hypertension	Yes vs. No	10	1083	2.31 [1.68, 3.18]	47.10%
Cardiovascular disease	Yes vs. No	7	906	2.76 [1.39, 5.45]	25.70%
Chronic obstructive pulmonary disease	Yes vs. No	5	623	3.56 [1.33, 9.54]	0%
**Vital Signs**					
Respiratory rate (per min)	Continuous	2	70	5.29 [2.56, 8.01]	42.80%
Symptoms					
Fever	Yes vs. No	10	2032	2.11 [1.11, 4.02]	64.20%
Dyspnea	Yes vs. No	8	977	8.83 [2.82, 27.67]	79.10%
**Laboratory Findings**					
**Blood routine**					
White blood cell count (×10^9/L)	Continuous	7	595	1.76 [0.31, 3.22]	88.70%
Lymphocytes count (×10^9/L)	Continuous	6	563	-0.42 [-0.52, -0.33]	11.50%
Neutrophils count (×10^9/L)	Continuous	2	159	2.92 [-1.33, 7.17]	93%
**Biochemical indicators**					
Albumin (g/L)	Continuous	4	323	-5.74 [-7.94, -3.54]	62.80%
Alanine aminotransferase (IU/L)	Continuous	4	189	16.97 [2.18, 31.76]	89.10%
Aspartate aminotransferase (IU/L)	Continuous	4	229	20.60 [6.81, 34.40]	86.90%
Lactate dehydrogenase (umol/L)	Continuous	3	120	3.93 [2.01, 5.86]	0%
Creatinine (umol/L)	Continuous	5	422	8.32 [-1.63, 18.28]	62.10%
**Inflammatory biomarkers**					
C-reactive protein (SMD)	Continuous	5	463	2.11 [0.71, 3.51]	96.10%
Procalcitonin (ng/ml)	Continuous	5	455	0.16 [0.04, 0.28]	77.80%
**Myocardial enzymes**					
Lactate dehydrogenase (SMD)	Continuous	5	291	2.0 [1.20, 2.80]	81.10%
Creatine kinase (IU/L)	Continuous	3	213	23.55 [17.08, 30.02]	30.80%
**Coagulation**					
D-dimer (mg/L)	Continuous	5	396	0.67 [0.02, 1.32]	72%

### Main results of patient characteristics, comorbidities, vital signs, symptoms and laboratory findings

A random-effects model (*I^2^*=54.4%) was used on 6 articles [7, 9, 11, 17, 24, 25] involving 681 patients to analyze the correlation between age and the severity of COVID-19 in patients. Compared with non-severe patients, the average age of severe patients was higher (WMD=11.89[8.63, 15.14]) than that of mild patients. Ten studies [7, 9, 11, 14, 15, 17, 19, 23–25] on gender included 1494 patients suggesting that men were more likely to have severe pneumonia than women (OR=1.61[1.25, 2.07]). Only two studies [18, 25] with 79 patients pointed out that the BMI values of severe patients were higher than those of non-severe patients (WMD=3.38[0.07, 6.69]). A fixed-effects model was applied on 3 articles [7, 14, 25] involving 412 patients to evaluate the risk factors of smoking. The results suggested that there was no significant correlation between smoking and severe pneumonia (OR=1.4[0.65, 3.01]).

We noted that patients with the following underlying diseases were more likely to develop severe COVID-19 conditions: diabetes mellitus (DM): OR=3.04[2.01, 4.60], hypertension (HTN): OR=2.31[1.68, 3.18], coronary heart disease (CHD): OR=2.76[1.39, 5.45], chronic obstructive pulmonary disease (COPD): OR=3.56[1.33, 9.54]. All the results were low heterogeneous (*I^2^*<50%). With respect to respiratory function, 2 articles [13, 25] (consisting of 70 patients in total) proved that patients with severe COVID-19 breathed faster (WMD=5.29[2.56, 8.01], *I^2^*=42.8%). Both fever (11 articles [7, 9, 13–17, 19, 22, 24, 25]) and dyspnea (8 articles [7, 9, 13, 14, 16, 17, 19, 24]) were risk factors for severe COVID-19 (Fever: OR=2.11[1.11, 4.02]; Dyspnea: OR=8.83[2.82, 27.67]).

In terms of laboratory parameters, we detected that severe patients had higher white-cell counts (WMD=1.76[0.31, 3.22]), and lower lymphocyte proportions (WMD=-0.42 [-0.52, -0.33]). Compared with non-severe patients, severe patients had higher levels of alanine aminotransferase (ALT), aspartate aminotransferase (AST), and total bilirubin (Tbil) (ALT: WMD=16.97[2.18, 31.76]), AST: WMD=20.60[6.81, 34.40], Tbil: WMD=3.93[2.01, 5.86]). Notwithstanding, values for creatinine (Cr) and neutrophil count (N count) were similar in both severe and mild cases (Cr: WMD=8.32[-1.63, 18.28], N count: WMD=2.92[-1.33, 7.17]. Levels of C-reactive protein (CRP) and procalcitonin (PCT) were more elevated in severe patients than in non-severe patients (CRP: SMD=2.11[0.71, 3.51], PCT: WMD=0.16[0.04, 0.28]). Furthermore, we found out that compared with non-severe patients, lactate dehydrogenase (LDH), creatine kinase (CK) and D-dimer (D-D) increased significantly in severe patients (LDH: SMD=2.0[1.20, 2.80], CK: WMD=23.55[17.08, 30.02], D-D: WMD=0.67 [0.02, 1.32]), but decreased in albumin (ALB, WMD=-5.74 [-7.94, -3.54]).

### Quality assessment

The quality of the included studies was assessed using the observational study quality evaluation criteria recommended by the AHRQ. All studies contained complete data sources, inclusion and exclusion criteria, and reasonable control of confounding factors. Nonetheless, only a few studies reported their quality control and management of missing data (See Supplementary Table 2).

### Publication bias

**Figure 4:**
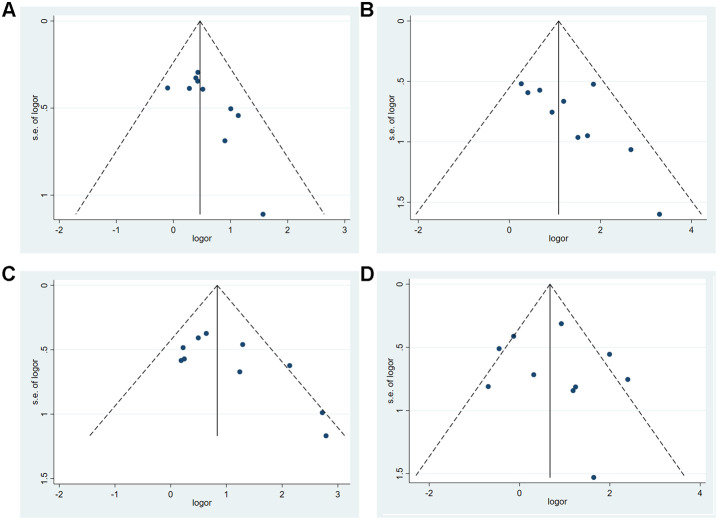
**The funnel-plots of (A) Gender, (B) Diabetes, (C) Hypertension, (D) Fever.**

We only drew funnel-plots for outcome indicators with more than 10 studies, and judged the publication bias of the results by observing the symmetry of the funnel-plots (See Figure 4). The funnel-plots were roughly symmetrical, indicating that publication bias was negligible. We did not only include officially published studies, but also literature published in medRxiv, Preprints and bioRxiv, as long as they met our inclusion criteria. Thus, the publication bias was low.

## DISCUSSION

COVID-19 is a new disease and there is limited information regarding risk factors for developing severe illness. Based on the analysis of available studies, risk factors for progressing to severe illness from COVID-19 include old adults, male gender, high BMI, comorbidities (DM, HTN, CHD, COPD), higher respiratory rate, fever, dyspnea, higher levels of white-cell counts, ALT, AST, Tbil, CRP, PCT, LDH, CK, D-D, and lower lymphocyte counts. To our knowledge, this is the first systematic review and meta-analysis summarizing the characteristics of patients severely infected with SARS-CoV-2.

At present, no effective drugs for the SARS-CoV-2 infection has been identified and clinical trials for the vaccine are still underway. Current clinical management includes infection prevention and control measures, together with supportive care, including supplemental oxygen and mechanical ventilatory support when indicated. Based on Chinese experience, mild patients and those who have been identified to have close contact with COVID-19 patients are managed by isolation and follow-up, which are were proven to be sufficient to manage the disease in most of these cases [26–28]. Nevertheless, intensive care and aggressive treatment modalities are needed for severe patients.

Guan et al [6] reported that the median (IQR) age of critically ill patients was 52.0 (40.0–65.0), which was older than that of non-critically ill patients whose median (IQR) age was 45.0 (34.0–57.0). Similarly in our review, we discovered that critically ill patients were older than non-critically ill patients, and most patients are male. Moreover, the presence of coexisting illnesses (such as DM, HTN, CHD, COPD) was more common among patients with severe COVID-19 than those with non-severe COVID-19. We know that the elderly with chronic diseases, especially those with diabetes mellitus usually have a high blood glucose status for long periods of time, and their immunity against infection is impaired [29]. They are at high risk for various infections, and once infected, they can easily develop severe illness. The lower sensitivity of women to viral infections may be due to the protection from X chromosomes and sex hormones, which play an important role in innate and adaptive immunity [30]. Thence, we believe that elderly male patients with underlying diseases are at high risk for severe COVID-19 and require special attention from clinicians. In addition, our study also found out that patients with high BMIs were more likely to develop severe pneumonia, which may be related to the high expression of angiotensin-converting enzyme 2 (ACE2) in obese patients [31]. ACE2 is a high affinity binding receptor for SARS-CoV-2. As adipose tissue expresses more ACE2 receptors [31], this may explain why obese patients are more susceptible to SARS-CoV-2 infection. Thus obese people should be more careful during the COVID-19 epidemic.

The symptoms of COVID-19 infection include fever, cough, sputum production, sore throat, fatigue or myalgia, dyspnea, nausea or vomiting, diarrhea, chills and headache. In most studies, fever and dyspnea occurred more frequently in critically ill patients [6, 8, 9], which is consistent with our study. Regarding vital signs, we observed that critically ill patients usually breathed faster than non-critically ill patients, which might be due to the low level of arterial oxygen saturation caused by lung injury in these patients. A study [25] with 49 individuals demonstrated that there was no considerable difference in the pulse rate of severe and non-severe patients (90.6±10.3 vs. 93.8±13.7, *p*=0.440), which is consistent with the findings of Fang et al [24]. Meanwhile, the Fang’s study [24] also concluded that the blood pressure of severe patients seemed to be higher than that of non-severe patients [SBP: 133.3±16.5 vs. 121.2±9.5, *p*<0.001; DBP: 83.3±11.7 vs. 74.2±9.5, p<0.001]. While the study by Xiang et al [25] stipulated that there was no significant difference in systolic blood pressure (*p*=0.769), but the diastolic blood pressure was lower (69.1±10.8 vs. 80.7± 12.7, *p*= 0.013) compared with non-severe patients.

As for laboratory tests, patients with severe disease had more prominent laboratory abnormalities (including lymphocytopenia, hypoalbuminemia, elevated levels of ALT, AST, Tbil, LDH, and CK) than those with non-severe disease. Many previous studies have reported lymphocytopenia in COVID-19 patients [32], as SARS-CoV-2 particles could damage the cytoplasmic components of lymphocytes and cause apoptosis [33]. In this meta-analysis, we realized that SARS-CoV-2 viral particles could damage other organs in addition to the lungs, as indicated by the elevated levels of ALT, AST, Tbil, LDH, and CK. As it is well-known, the coronavirus (CoV) is a pathogen that can infect the respiratory, gastrointestinal, hepatic, and central nervous systems of humans and many other wild animals [34, 35]. The homology of the genomic sequences of SARS-CoV-2 and SARS-CoV is 85% at the nucleotide level, which is extremely high [36, 37]. The latest pathology reports [38] revealed that the pathological manifestations of SARS-CoV-2 and SARS-CoV are similar, chiefly manifesting as acute respiratory distress syndrome (ARDS). SARS-CoV-2 has been proven to infect humans’ respiratory epithelial cells by binding to the angiotensin-converting enzyme 2 receptors on human cells [39, 40]. Additionally, several studies [31, 41] have outlined that in the human body, levels of ACE2 RNA were higher in heart, kidney, intestinal tract, gallbladder, adipose tissue and testicles than in the lungs. This finding also explains the reason multi-organ dysfunction is prone to occur in critically ill patients.

Levels of inflammatory biomarkers were high in COVID-19 patients and might have contributed to disease severity as well as mortality. In this study, we noted that CRP and PCT increased in severely ill patients. Some research also reported elevated levels of ESR, IL-6, and IL-10 in critically ill patients [10]. It is considered that multiple cytokines are secreted after infection with microorganisms, which might induce a strong inflammatory response and damage the immune system. This phenomenon illustrates that critically ill patients may have more severe systemic inflammatory response, so attention should be paid to anti-inflammatory treatment [25].

On March 11, the WHO announced that it could use the term “Pandemic” to describe the epidemic form of COVID-19 [42]. This implies that the WHO officially recognizes that COVID-19 has become a global pandemic. In order to further understand the risk factors of critically ill patients, we also pay close attention to the latest overseas reports on COVID-19. From February 12 to March 28, 2020, a first large data report [43] from the NCP in the United States stipulated that among 7162 diagnosed patients, 2,692 (37.6%) patients had one or more underlying diseases. The most common underlying diseases include diabetes (10.9%), cardiovascular diseases (9.0%) and chronic lung disease (9.2%). The study also concluded that the elderly had a high risk of developing severe illness, which is consistent with our study. Another study from Italy [44] also showed that a high proportion of elderly patients were infected with COVID-19, and most fatalities were older male patients with underlying health conditions.

## Limitations

There are some limitations in our study. Firstly, all included studies were cross-sectional, precluding the possibility to establish inferences regarding causality. Secondly, there might be discrepancies in populations, risk factors analyzed, lengths of follow-up, and statistical methods leading to a certain extent of study heterogeneity. Thirdly, despite the fact that this study reviewed the risk factors for progressing to COVID-19 disease, we were not able to assess the amplitude of each risk factor and develop a risk model accordingly.

## CONCLUSIONS

The severity of SARS-CoV-2 pneumonia has exerted immense pressure on the intensive care resources of hospitals, especially in some developing countries lacking medical staff and health resources. Consequently, early identification of patients at risk for severe illness may reduce the mortality rate by providing them prompt treatment.

## MATERIALS AND METHODS

This meta-analysis was performed in accordance with PRISMA-2009 (Preferred Reporting Items for Systematic Reviews and Meta-analyses) [45] and MOOSE (Meta-analysis of Observational Studies in Epidemiology) guidelines [46].

### Search strategy

Relevant studies were searched from both Chinese and English electronic bibliographic databases, including China National Knowledge Infrastructure (CNKI), Wanfang Database, Weipu Database, Chinese Biomedicine Literature Database (CBM-SinoMed), PubMed, Embase, Cochrane Central Register and Web of Science from inception to 8 March 2020. MeSH terms for COVID-19 and corresponding synonyms were included into the searching strategy. We limited our investigation to human-subjects. Reference lists of retrieved articles were also reviewed to further identify potentially relevant studies. Searching methodologies were independently conducted by two reviewers (Lizhen Xu and Yaqian Mao) and disagreements were settled by discussion. Institutional review board approval and informed consent were not obtained given that the study is a systematic review of the literature, thus we limited our study to published information and did not engage with any human subjects.

### Inclusion criteria

The inclusion criteria were as follows: (i) Prospective or retrospective original reports; (ii) All of the patients were diagnosed with COVID-19; (iii) Characteristics of severe and mild cases were documented; and (iv) Complete medical records were available for data extraction.

Definition of severe COVID-19 illness [47]: Severe COVID-19 illness was designated exclusively when patients met at least one of the following criteria: 1) Respiratory distress with respiratory frequency≥30 breaths/min; 2) Pulse Oximeter Oxygen Saturation≤93% at rest; 3) Oxygenation index (Partial pressure of oxygen in arterial blood/Inspired oxygen fraction, PaO_2_/FiO_2_)≤300mmHg (1mmHg=0.133 kPa). At high altitudes (above 1000 meters), PaO_2_/FiO_2_ should be corrected according to the following principle: PaO_2_/FiO_2_×[Atmospheric Pressure (mmHg)/760]. Cases where pulmonary imaging revealed that the lesions progressed by more than 50% within 24-48 hours should be managed as critically ill patients.

### Data extraction

The following characteristics were extracted from the selected studies: authors, sample size, region, and study period. In addition, the following potential risk factors were recorded independently: patient demographic characteristics, comorbidities, vital signs, symptoms, and laboratory findings. Data extraction was accomplished independently by two reviewers (LX and YM). Any divergence of opinions was resolved by discussion. In order to minimize data duplication, when two or more studies shared identical information, whenever sampling periods overlapped and patients were from the same geographic region, the ones with the largest population were input. Continuous variables were expressed as medians and interquartile ranges (IQR) or simple ranges in some studies. However, the standard deviation and mean value were not estimated due to inaccuracy.

### Statistical analysis

For categorical variables, analysis was performed by calculating the odds ratio (OR) with 95% confidence interval (95%CI). For continuous outcomes, weighted mean difference (WMD) and standardized mean difference (SMD) were calculated with the corresponding 95%CI. Heterogeneity was assessed using the *I^2^* test, with *I^2^*>50% indicating the existence of heterogeneity. In the occurrence of significant heterogeneity, a random effect model (DerSimonian-Laird method) was used to calculate the pooled effect; Otherwise, the fixed model (Mantel-Haenszel method) was used instead. Possible publication bias was evaluated via observing the symmetry characteristics of funnel-plots. If the number of included studies in each outcome was<10, the funnel-plots was not carried out due to limited power [48]. Data analysis was undertaken using STATA, version 15.0.

### Quality assessment

The observational study quality evaluation criteria recommended by the American Agency for Healthcare Research and Quality (AHRQ) were used to analyze the study’s quality. These criteria consisted of 11 items, composed of subjects selection, research quality control and data processing. Each question will be answered with either “yes”, “no” or “unclear.”

## Supplementary Material

Supplementary Table 1

Supplementary Table 2
