# Supplementary material for: Risk factors for 2019 novel coronavirus disease (COVID-19) patients progressing to critical illness: a systematic review and meta-analysis
Source: Aging (Albany NY). 2020 Jun 23;12(12):12410–21. doi: 10.18632/aging.103383 (PMC7343456; doi:10.18632/aging.103383)
Supplement: Supplementary Table 1 [file aging-12-103383-s001..docx]

**Supplementary Table 1. Summary the characteristics of 20 studies that described the risk factors with COVID-19 patients^1^**

| Study | Size  (n) | Region | Study period | Outcomes：Characteristics of severe patients | | | | |
| --- | --- | --- | --- | --- | --- | --- | --- | --- |
|  |  |  |  | Patient  features | Comorbidities | Vital Signs | Symptoms | Laboratory Findings |
| Guan  WJ^[10]^ | 1099 | 552 hospitals in 30 provinces | 2019.12.11-  2020.01.29 | **Age↑**, Sex, **Smoking↑**, Exposure | **DM↑, COPD↑, CHD↑,**  **Hypertension↑**, CKD, Cancer, Cerebrovascular disease, Hepatitis, Immunodeficiency, | **T↑** | Fever, Cough, Sputum production, Sore throat, Fatigue or Myalgia, **Dyspnea↑**, Nausea or vomiting, Diarrhea, Chills, Headache | Blood routine **(WBC↓**, **LYM↓**, **PLT↓**, **Hb↓)**, Biochemical indicators **(ALT↑**, **AST↑**, **Cr↑)**, Myocardial enzymes, cTnI **(LDH↑)**, Coagulation **(D-dimer↑)**, Minerals **(Potassium↓)** |
| Yuan  J^[11]^ | 223 | Chong qing  Public Health  Medical Center | 2020.01.24-  2020.02.23 | **Age↑**, Sex, Smoking, Exposure | **DM↑**, Hypertension, CHD, COPD, Liver disease, HIV | **T↑, R↑** | Fever, Cough, Sputum production, **Dyspnea↑,** Fatigue or Myalgia, Headache, Diarrhea | Blood routine **(LYM↓)**, Biochemical indicators **(ALB↓**,**TB↓**, **ALT↑**, **AST↑)**, Inflammatory biomarkers **(PCT↑)**, Myocardial enzymes, cTnI **(CK↑**, **LDH↑)**, Coagulation (D-dimer) |
| Wan  Q^[12]^ | 153 | Chong qing  Public Health  Medical Center | 2020.01.26-  2020.02.05 | Age, Sex, Exposure | **DM↑**, Hypertension, **Respiratory disease↑**, Liver disease | NR | **Fever↑**, **Sputum production↑**, **Dyspnea↑**,**Myalgia↑**, **Fatigue↑**, Chills, Diarrhea, Abdominal pain, nasal discharge, Cough, | Inflammatory biomarkers **(CRP↑)**, Lymphocyte subsets **(CD3↓**, **CD4+↓**, **CD8+↓)**，Myocardial enzymes, cTnI (LDH), Coagulation **(D-dimer↑)** |
| Chen YJ ^[13]^ | 143 | Chongqing Three Gorges Central Hospital | 2020.01.23-  2020.02.08 | Age, Sex, Exposure | DM, Hypertension, CHD, Liver disease, Respiratory disease | NR | **Fever↑**, Cough, Sore throat, **Dyspnea↑**, Myalgia, Fatigue, Headache, Chills | Blood routine **(LYM↓)**, Inflammatory  Biomarkers **(CRP↑**, **IL-6↑**,**PCT↑)**, Myocardial enzymes, Coagulation (D-dimer) |
| Wan  SX^[25]^ | 123 | Chongqing Three Gorges  Central Hospital | 2020.01.26-  2020.02.04 | NR | **DM↑**, **CKD↑**，**CHD↑**，Hypertension, COPD, Cerebrovascular disease, | NR | Fever, Dry cough, **Fatigue↑**, Stethalgia, Diarrhea | Lymphocyte subsets **(CD4↓**, **CD8↓)**, Cytokines status **(IL-6↑**, **IL-10↑)** |
| Chen  C^[14]^ | 150 | Wuhan Tongji hospital | 2020.01-  2020.02 | **Age↑**, Sex **(male↑)** | DM, **Hypertension↑**, **CHD↑** | NR | NR | Biochemical indicators **(Cr↑)**, Myocardial enzymes, cTnI **(NT-proBNP↑**, **cTnI↑)**, Inflammatory biomarkers **(CRP↑)** |
| Chen,  L^[28]^ | 29 | Wuhan Tongji hospital | 2020.01.14-  2020.01.29 | NR | NR | NR | NR | Blood routine, Cytokines status (**IL-2R ↑**， **IL-6 ↑)**, Myocardial enzymes, cTnI |
| Chen  G^[15]^ | 21 | Wuhan Tongji hospital | 2019.12.19-  2020.01.27 | **Age↑,** Sex, Exposure | DM, Hypertension | **R↑**,T, HR, SBP | Fever, Cough, Fatigue, Myalgia, Sputum production, Headache, Diarrhea, **Dyspnea↑** | Blood routine **(WBC↑**, **NEU↑**, **LYM↓)**, Biochemical indicators **(ALT↑**, **AST↑**, **ALB↓**, **BUN↑)**, Myocardial enzymes, cTnI **(LDH↑)**, Inflammatory biomarkers **(CRP↑**, **Ferritin↑)**, Coagulation **(APTT↓**, **D-dimer↑)** |
| Zhang  JJ^[16]^ | 140 | No.7 hospital of Wuhan | 2020.01.16-  2020.02.03 | **Age↑**, Sex, Smoking, Exposure | DM, Hypertension, liver disease, CHD, CKD, COPD, Cerebrovascular disease, | NR | Fever, **Cough↑**, Fatigue, dyspnea, **Nausea↑**, Abdominal pain, Diarrhea | Blood routine **(WBC↑**, **LYM↓**), Biochemical indicators, Inflammatory biomarkers **(CRP↑**, **PCT↑)**, Coagulation **(D-dimer↑)** |
| Liu  YL^[17]^ | 109 | The Central Hospital of Wuhan | 2020.01.02-  2020.02.01 | **Age↑**, Sex | **DM↑**, Hypertension, COPD, **CHD↑**, **CKD ↑**, Cerebrovascular disease, | NR | Fever, Dry cough, **Fatigue↑**, Stethalgia, Diarrhea | Blood routine **(NEU↑**, **LYM↓)**, Biochemical indicators **(BUN↑)**, Inflammatory biomarkers **(CRP↑**, **PCT↑**, **Lactate↑)**, Coagulation **(Fibrinogen↑**, **D-dimer↑)**, Myocardial enzymes, **cTnI (LDH↑)** |
| Xiong  J^[26]^ | 89 | Renmin Hospital of Wuhan University | 2020.01.17-  2020.02.20 | NR | DM, Hypertension, Cancer, Cerebrovascular disease, Liver disease, renal disease | NR | **Fever↑**, **Cough↑**, Sputum production, **Dyspnea↑**, Fatigue, Myalgia, Headache, Diarrhea, Abdominal pain,  Nausea, vomiting, Sore throat | Blood routine **(LYM↓)**, Biochemical indicators **(Cr↑**, **Urea↑)**, Inflammatory biomarkers **(CRP↑)**, Myocardial enzymes, cTnI **(cTnI↑)** |
| Liu J^18]^ | 40 | Wuhan Union Hospital | 2020.01.05-  2020.01.24 | **Age↑**, Sex, Exposure | DM,  **Hypertension↑**, Cancer | T | Fever, Chill, Fatigue, **Myalgia↑**, Fatigue, **Sputum production↑**, Pharyngalgia, Dizziness or Dizziness, Chest pain, Dyspnoea, Diarrhoea, **Nausea↑**, Vomiting | Blood routine **(WBC↑**, **NEU↑**, **LYM↓)**, Biochemical indicators **(TBil↑**, **ALT↑**, **AST↑)**, Inflammatory biomarkers **(CRP↑**, **Ferritin↑**, **SAA↑)**, Myocardial enzymes, cTnI **(LDH↑**, **CK↑)**, Coagulation **(D-dimer↑**, **Fibrinogen↑)** |
| Liu M^[19]^ | 30 | The Affiliated Hospital ofJianghanUniversity,Wuhan | 2020.01.10-  2002.01.31 | **BMI↑** | NR | NR | **Duration of fever↑** | Blood routine **(WBC↑**, **LYM↓)**, Biochemical indicators **(ALB↓**, **ALT↑**, **AST↑)** , Myocardialenzymes, cTnI **(LDH↑)**, Coagulation **(D-dimer↑)** |
| Tian  SJ^[20]^ | 262 | The designated hospitals in Beijing | 2020.01.20-  2020.02.10 | **Age↑,** Sex, Exposure | NR | T, R | Fever, Cough, Fatigue, **Dyspnea↑**, Headache | NR |
| Liu  JY ^[21]^ | 61 | Beijing Ditan Hospital | 2020.01.13-  2020.01.31 | **Age↑**, Sex, smoking, Exposure | DM, Hypertension, CHD, COPD, | T,**R↑**  SBP, DBP | Fever, **Dyspnea↑**,Cough, **Sputum production↑**, Fatigue, Headache, Chill, Nausea or vomiting, Diarrhea, Sore throat, Chest pain | Blood routine, Biochemical indicators, Inflammatory biomarkers, Coagulation, Minerals |
| Wen  K^[29]^ | 46 | The Fifth Medical Center of Chinese PLA General Hospital, Beijing | 2020.01.20-  2020.02.08 | NR | NR | NR | NR | Inflammatory biomarkers **(CRP↑**, **IL-6↑**, **ESR↑**, **Ferritin↑**, **Lactate↑)**, Lymphocyte subsets **(CD8+↓)** |
| Dai  ZH^[27]^ | 918 | Hunan Province | 2020.01.21-  2020.02.13 | NR | NR | NR | Fever, Cough, Myalgia, Fatigue, Dyspnea, Sputum production, Headache, Chills, Diarrhea, | Blood routine **(LYM↓**, **NEU%↑)** |
| Cai QX^[22]^ | 298 | The Third People's Hospital of Shenzhen | 2020.01.11-  2020.02.06 | **Age↑**, Sex, BMI | **Basic diseases↑** | NR | **Duration of fever↑** | Blood routine **(LYM↓)**, Inflammatory biomarkers **(CRP↑**, **IL-6↑**，**ESR↑)**, Myocardial enzymes, cTnI **(CK↑**, **LDH↑**, **Mb↑)**, Coagulation **(D-dimer↑)** |
| Fang  XW^[23]^ | 79 | Anhui Provincial Hospital | 2020.01.22-2020.02.18 | **Age↑**, Sex  **(male↑)**, Exposure | DM, **Hypertension↑**, Respiratory disease, CHD, **Cerebrovascular disease↑**, Liver disease, renal disease, Cancer | T, P, R, **SBP↑**  **DBP↑** | Fever, Cough, Myalgia or Fatigue, Dyspnea, Sputum production, Dizziness or Headache, Poor appetite, Sore throat, Diarrhea, Nausea, vomiting | Blood routine **(WBC↑**, **LYM↓**, **NEU%↑)**, Biochemical indicators **(ALB↓**, **BUN↑**), Inflammatory biomarkers **(CRP↑)**, Myocardial enzymes, cTnI **(LDH↑**, **cTnI↑)**, Coagulation **(D-dimer↑)** |
| Xiang  TX^[24]^ | 49 | First affiliated hospital of Nanchang University | 2020.01.21-  2020.01.27 | **Age↑**, Sex,BMI, Smoking, Exposure | DM, Hypertension, Liver disease | T, P, **R↑**, SBP, **DBP↓** | Fever, Cough, Sputum production, Dyspnea, Myalgia, Fatigue, Headache, Diarrhea, Sore throat, Chills | Blood routine **(WBC↑**, **LYM↓)**, Biochemical indicators **(ALB↓**, **TBil↑**, **ALT↑**, **AST↑)**, Minerals **(Calcium↓)**, Inflammatory biomarkers **(ESR↑**, **CRP↑**, **SAA↑)**, Myocardial enzymes, cTnI **(LDH↑)**, Lymphocyte subsets **(CD3↓**, **CD4↓**, **CD8↓)** |

**^1^DM**：Diabetes mellitus, **CKD**:Chronic renal disease, **CHD**:Coronary heart disease, **T**:Temperature, **R**: Respiratory, **P**: Pulse, **SBP**:Systolic blood pressue, **DBP**:Diastolic blood pressure, **ALT**: alanine aminotransferase, **AST**, aspartate aminotransferase,**TBil**: total bilirubin, **LDH**: lactate dehydrogenase, **CK**: creatine kinase, **D-D**: D-dimer, **WBC count**: White blood cell count, **LYM**: Lymphocytes, **NEU**: Neutrophils, **ALB**: Albumin, **CRP**: C-reactive protein, **PCT**: Procalcitonin, **SAA**: Serum amyloid A, **APTT:** Activated partial thromboplastin time**,BUN**: Blood urea nitrogen, **Cr**: Creatinine, **NT-proBNP**: N-terminal protype B natriuretic peptide, **cTnI**: Cardiac troponin I, **CD3、CD4、CD8**：T lymphocyte subsets，**IL**: Interleukin，**Mb**: Myoglobin, **NR**：No report, **↑:**Indicate that patients with critically ill are more obvious than those with non-critically ill, **↓:**Indicates that the critically ill patientsare not obvious than the non-critically ill patients.
