# Supplementary material for: Risk factors for 2019 novel coronavirus disease (COVID-19) patients progressing to critical illness: a systematic review and meta-analysis
Source: Aging (Albany NY). 2020 Jun 23;12(12):12410–21. doi: 10.18632/aging.103383 (PMC7343456; doi:10.18632/aging.103383)
Supplement: Supplementary Table 2 [file aging-12-103383-s002..pdf]

Supplementary Table 2. Quality assessment: Cross-sectional/prevalence study quality according to AHRQ.

Study	1	2	3	4	5	6	7	8	9	10	11
Guan WJ	Yes	Yes	Yes	Unclear	Unclear	Yes	Yes	Yes	No	No	No
Yuan Jing	Yes	Yes	Yes	Unclear	Unclear	No	No	No	No	No	No
Wan Qiu	Yes	Yes	Yes	Unclear	Unclear	No	No	No	No	No	No
Xiao Kaihu	Yes	Yes	Yes	Unclear	Unclear	Yes	No	Yes	No	No	No
Suxin Wan	Yes	Yes	Yes	Unclear	Unclear	No	No	Yes	No	No	No
Chen, C	Yes	Yes	Yes	Unclear	Unclear	No	No	Yes	No	No	No
Chen Lei	Yes	Yes	Yes	Unclear	Unclear	No	No	No	No	No	No
Chen Guang	Yes	Yes	Yes	Unclear	Unclear	Yes	No	Yes	No	No	No
Zhang Jin-Jin	Yes	Yes	Yes	Unclear	Unclear	Yes	No	Yes	No	No	No
Liu Yanli	Yes	Yes	Yes	Unclear	Unclear	Yes	Yes	Yes	No	Yes	No
Xiong Juan	Yes	Yes	Yes	Unclear	Unclear	No	No	No	No	No	No
Liu Jing	Yes	Yes	Yes	Unclear	Unclear	Yes	No	Yes	No	No	No
Liu min	Yes	Yes	Yes	Unclear	Unclear	Yes	No	Yes	No	No	No
TianSijia	Yes	Yes	Yes	Unclear	Unclear	Yes	No	Yes	No	No	No
Jingyuan Liu	Yes	Yes	Yes	Unclear	Unclear	Yes	No	Yes	No	No	No
Wen Ke	Yes	Yes	Yes	Unclear	Unclear	No	No	No	No	No	No
Dai Zhihui	Yes	Yes	Yes	Unclear	Unclear	No	No	No	No	No	No
CaiQingxian	Yes	Yes	Yes	Unclear	Unclear	Yes	No	Yes	No	No	No
Fang Xiaowei	Yes	Yes	Yes	Unclear	Unclear	No	No	No	No	No	No
Xiang Tianxin	Yes	Yes	Yes	Unclear	Unclear	No	No	No	No	No	No

1) Define the source of information; 2) List inclusion and exclusion criteria for exposed and unexposed subjects (cases and controls) or refer to previous publications;3) Indicate time period used for identifying patients;4) Indicate whether or not subjects were consecutive if not population-based;5) Indicate if evaluators of subjective components of study were masked to other aspects of the status of the participants;6) Describe any assessments undertaken for quality assurance purposes (e.g., test/retest of primary outcome measurements);7) Explain any patient exclusions from analysis;8) Describe how confounding was assessed and/or controlled.;9) If applicable, explain how missing data were handled in the analysis;10) Summarize patient response rates and completeness of data collection;11) Clarify what follow-up, if any, was expected and the percentage of patients for which incomplete data or follow-up was obtained.
